# Unexpected perinatal death caused by an occult MTM1 mutation: a case report

**DOI:** 10.3389/fmed.2026.1660209

**Published:** 2026-03-24

**Authors:** Man-Man Zhu, Dong-Mei Li, Yi-Cheng Wu, Qiang Yao, Ming-Rong Qie, Wei-Wei Sun

**Affiliations:** 1 Department of Obstetrics and Gynecology, West China Second University Hospital, Sichuan University, Chengdu, China; 2 Key Laboratory of Birth Defects and Related Diseases of Women and Children (Sichuan University), Ministry of Education, Chengdu, China

**Keywords:** case report, MTM1, stillbirth, whole exome sequencing testing, X-linked centronuclear myopathies

## Abstract

**Background:**

Genetic mutations can lead to miscarriages, perinatal deaths, and abnormalities in fetal development. Sometimes, the regular prenatal test cannot identify some rare diseases, but whole-exome sequencing can be performed. Whole-exome sequencing testing is not a routine prenatal test unless there is a family history or a history of adverse pregnancy due to a genetic disorder.

**Case presentation:**

We reported a case of stillbirth caused by a rare genetic disease, secondary to an MTM1 gene mutation. It was found to have no abnormality during childbirth; even electronic fetal monitoring showed no signs of fetal distress. However, delivery concluded with a stillborn birth, and the offspring having no crying, poor muscle tone, and pale skin. Due to the unclear cause of stillbirth, the patient requested further diagnostic genetic testing to identify a cause. The MTM1 gene hemizygous variant was detected by whole-exome sequencing.

**Conclusion:**

Stillbirth due to genetic mutations may not be detected by non-invasive prenatal testing or chromosome copy number variant analysis. Broad Next-Generation Sequencing, such as whole-exome sequencing, has the potential to identify genetic causes that are missed by non-invasive prenatal testing or chromosomal microarray. The indications for the whole-exome sequencing test for pregnant women may need further discussion.

## Background

According to National Vital Statistics Reports from the United States, a total of 46,876 fetal deaths after 20 weeks are caused by congenital malformations, deformations, and chromosomal abnormalities, accounting for 10.5% of births in 2018–2020 (1). Genetic mutations can lead to miscarriages, abnormalities in fetal development, or even perinatal deaths. Chromosome deletions or duplications that cause mortality can be detected by regular chromosomal microarray analysis (CMA). However, single-gene mutations are not detected by CMA, so broad/untargeted next-generation sequencing, such as whole-exome sequencing (WES), is needed. We present a case of stillbirth caused by a rare mutation in the MTM1 gene.

Mutations in the MTM1 gene can cause X-linked centronuclear myopathies (XLCNM, OMIM #310400), identified by Laporte et al. (2), also called X-linked myotubular myopathy(X-MTM), characterized by severe-to-mild muscle weakness.

Centronuclear myopathies (CNM) are congenital myopathies that have been described with both autosomal dominant (ADCNM, OMIM#160150) and autosomal recessive (ARCNM, OMIM#255200) inheritance (3–6).

In this case, we report a case of stillbirth caused by a rare genetic disease. We present the prenatal examination and clinical presentation, tracking the patient’s past medical history. We aim to enhance the understanding of the clinical features of genetic diseases and inform future diagnosis, treatment, and prevention of such diseases.

## Case presentation

**Figure 1:**
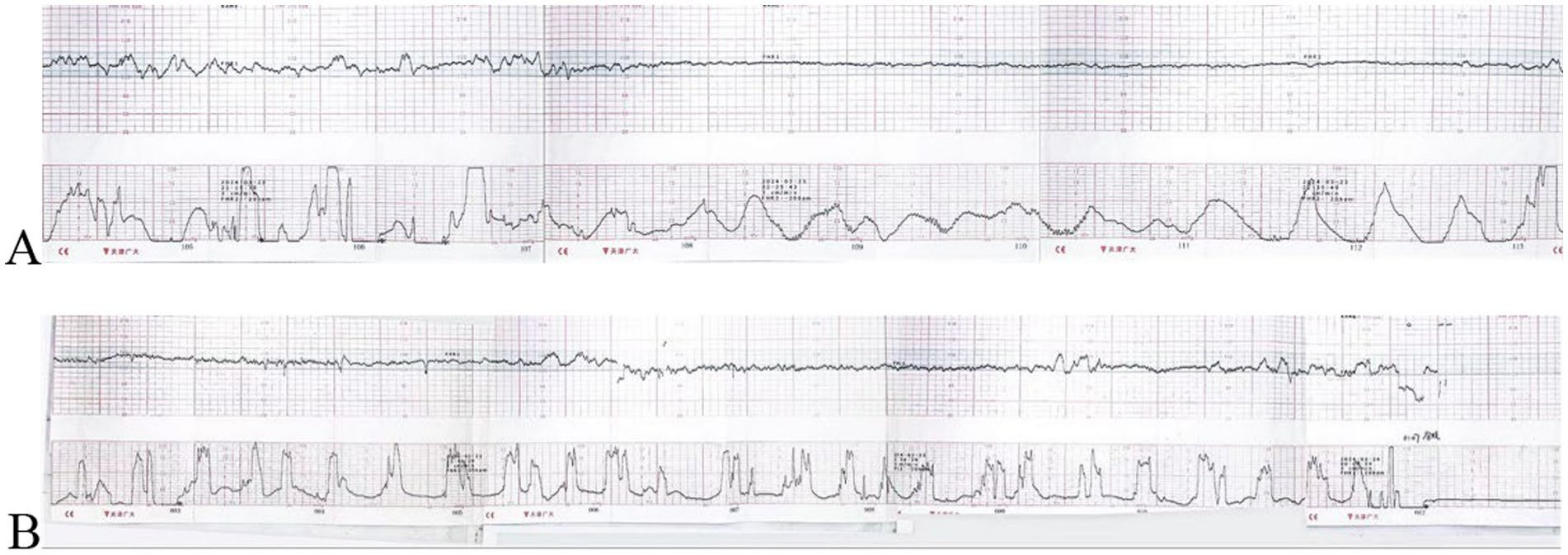
(**A)** Fetal monitoring in the first stage of labor (fetal heart rate above, uterine contractions below), category I. (**B)** Fetal monitoring in the second stage of labor, category I.

A 31-year-old nulliparous Han Chinese woman was admitted to our hospital in Sichuan, China, at 36 + 6 weeks of gestation due to spontaneous labor onset. She and her partner were both Han Chinese, with no known consanguinity. The family history was unremarkable, with no hereditary, neuromuscular, or congenital disorders reported. The non-invasive prenatal testing (NIPT) indicated low risk during her prenatal visit. At 32 + 4 weeks of gestation, the Doppler ultrasound showed polyhydramnios (a fluid depth of 9.3 cm and an amniotic fluid index of 27.4 cm) and the widening of the fetal left lateral ventricle of 1.11 cm. A magnetic resonance imaging (MRI) of the fetal brain revealed a width of 1.34 cm in the left lateral ventricle trigone area and 0.9 cm in the right area. After prenatal genetic counseling, CMA via amniocentesis was pursued and showed no significant chromosome deletions or duplications. The following Doppler ultrasounds continue to indicate polyhydramnios, with an amniotic fluid depth of 8.4 cm and an amniotic fluid index of 27.3 cm. During the inpatient time, electronic fetal monitoring showed no abnormal results during the first stage or second stage of labor (Figure 1). At birth, the newborn had no crying, poor muscle tone, and pale skin after delivery. Apgar scores were 0, 0, and 0 at 1, 5, and 10 min, retrospectively. We performed a variety of life-saving measures, including cardiopulmonary resuscitation, fluids, and epinephrine injections, but the patient did not regain spontaneous breathing. The stillborn male weighed 2230 grams, had a length of 49 cm, and showed no apparent deformities in appearance. Upon repeated inquiry into the patient’s family history, it was revealed that her grandmother had a history of two stillbirths of male infants. Due to the unclear cause of stillbirth, the clinician offered further diagnostic genetic testing.

**Figure 2:**
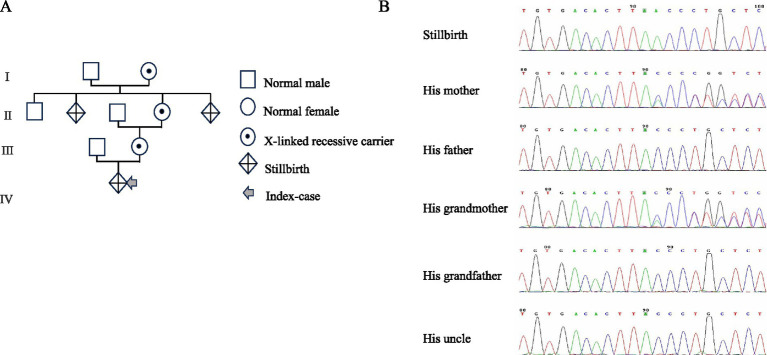
**(A)** Pedigree of the family. **(B)** Sanger sequencing results show a mutation in his mother and his grandmother, detected in stillbirth. The variant sites detected in patients with myotubular myopathy are at the same amino acid codon (c.594C>A, p.Y198*; c.594C>G, p.Y198*).

Regarding potential non-genetic causes, the results of the patient’s pregnancy and negative screening for immune-related antibodies, such as anticardiolipin antibodies and beta-2 antibodies, made stillbirth due to immune-related disorders unlikely. The absence of fever during pregnancy and labor, as well as the discharge from the uterine cavity and external auditory canal of the stillborn infant, was negative, decreasing the likelihood of intrauterine infection leading to stillbirth. Laboratory examination showed the alpha-fetoprotein (AFP) was 72.2 ng/mL, while the ABO blood type system, free antibody assay, and direct antiglobulin test were weakly reactive (trace positive); they were considered clinically insignificant as there was no evidence of fetal hydrops, laboratory evidence of hemolysis, or profound anemia. Furthermore, maternal–fetal transfusion syndrome was ruled out based on the absence of fetal red cells in the maternal circulation. Autopsy revealed a structurally normal fetal heart without congenital anomalies. The lungs showed no dilation of most alveoli, suggesting no signs of respiratory activity. Pedigree-based WES was conducted using the stillborn infant as the proband. To facilitate variant filtration and segregation analysis, DNA samples were also obtained from their mother, father, maternal grandmother, maternal grandfather, and a maternal uncle. The analysis identified a hemizygous nonsense mutation in the MTM1 gene on the X chromosome (chrX-150641333; c.594C>A, p.Y198*). Heterozygous variants were found only in the mother and maternal grandmother, with no genetic abnormalities identified in the other tested individuals (Figure 2).

Following the vaginal delivery, the patient received routine postpartum care and supportive psychological counseling to assist with the emotional impact of the perinatal loss. Her physical recovery was stable, and she was discharged on the third day of postpartum. At her 6-week follow-up visit at the postpartum clinic, her physical examination was unremarkable, and her overall recovery was satisfactory, with no remaining clinical concerns.

## Discussion

This case reports a gene mutation that cannot be detected by routine prenatal screening. The identified mutation (c.594C>A, p.Y198*) is a nonsense variant leading to a total loss of myotubularin function. While MTM1 mutations are heterogeneous, amino acid codon 198 is recognized in ClinVar (pathogenic) and HGMD as a recurrent site (hotspot) for lethal mutations. Specifically, both c.594C>A and c.594C>G transitions result in the same p.Y198* premature stop codon, which is consistently associated with the severe neonatal X-linked myotubular myopathy phenotype. This context explains the predictable lethality observed in this proband and his male relatives. XLCNM is a severe type of CNM in affected males and is characterized by severe muscle weakness, severe bulbar and respiratory involvement, polyhydramnios, and may decrease fetal movements during pregnancy (7–9). This clinical manifestation was also present in this report. It is estimated that at least 25% of males will die because of severe XLCNM within the first year of life, and few survivors reach adulthood (7). Males with mild or moderate XLCNM (20%) reach motor milestones faster than those with severe cases; many can walk independently and may live to adulthood. Females who are carriers of XLCNM are usually asymptomatic (10, 11). XLCNM also involves respiratory insufficiency, ophthalmoplegia/paresis, ptosis and facial weakness, external ophthalmoplegia, diaphragmatic atrophy leading to thinning, decreased liver function, fatal hepatic hemorrhage, and the disappearance of central nervous system reflexes (2, 4, 10, 12). In this case, XLCNM caused death.

## Conclusion

In this case, the genetic variants eventually caused fetal death. This case reports a stillbirth not detected by NIPT or CMA during pregnancy. The death of a newborn caused by a genetic mutation is an enormous harm to a family, especially to the patient, both mentally and physically. Subsequent whole-exome sequencing revealed the genetic cause. According to this case, preimplantation genetic diagnosis may be an option to avoid an abnormal pregnancy (13, 14).

## Data Availability

The original contributions presented in the study are included in the article/supplementary material, further inquiries can be directed to the corresponding author.
